
JOURNAL INFORMATION
==============================
NLM Title Abbreviation: Pharmacol Res Perspect
Journal ID: Pharmacol Res Perspect
NLM Journal ID: 101626369
Journal ID: PRP2

Pharmacology Research & Perspectives

EISSN: 2052-1707
Publisher: Wiley

ARTICLE INFORMATION
==============================
PMCID: PMC11474310
DOI: 10.1002/prp2.1097
Article ID: PRP21097
Article version: 1
Subjects: Issue Information, Issue Information

Issue Information

Electronic publication date: 2024 Feb 26
Publication date: 2024 Apr
Volume: 12
Issue: 2
Electronic Location ID: e01097
Copyright: © 2024 John Wiley & Sons Ltd, British Pharmacological Society and American Society for Pharmacology and Experimental Therapeutics
License: This is an open access article under the terms of the http://creativecommons.org/licenses/by/4.0/ License, which permits use, distribution and reproduction in any medium, provided the original work is properly cited.
License URL: https://creativecommons.org/licenses/by/4.0/

Figure count: 0
Table count: 0
Page count: 3

source-schema-version-number: 2.0
cover-date: April 2024
details-of-publishers-convertor: Converter:WILEY_ML3GV2_TO_JATSPMC version:6.4.9 mode:remove_FC converted:15.10.2024

==============================

### PR&P

### Pharmacology Research & Perspectives

Open Access

Editor-in-Chief
**Michael F. Jarvis, PhD, FBPhS**
Deputy Editor-in-Chief
**Jennifer Martin, FRACP, PhD**

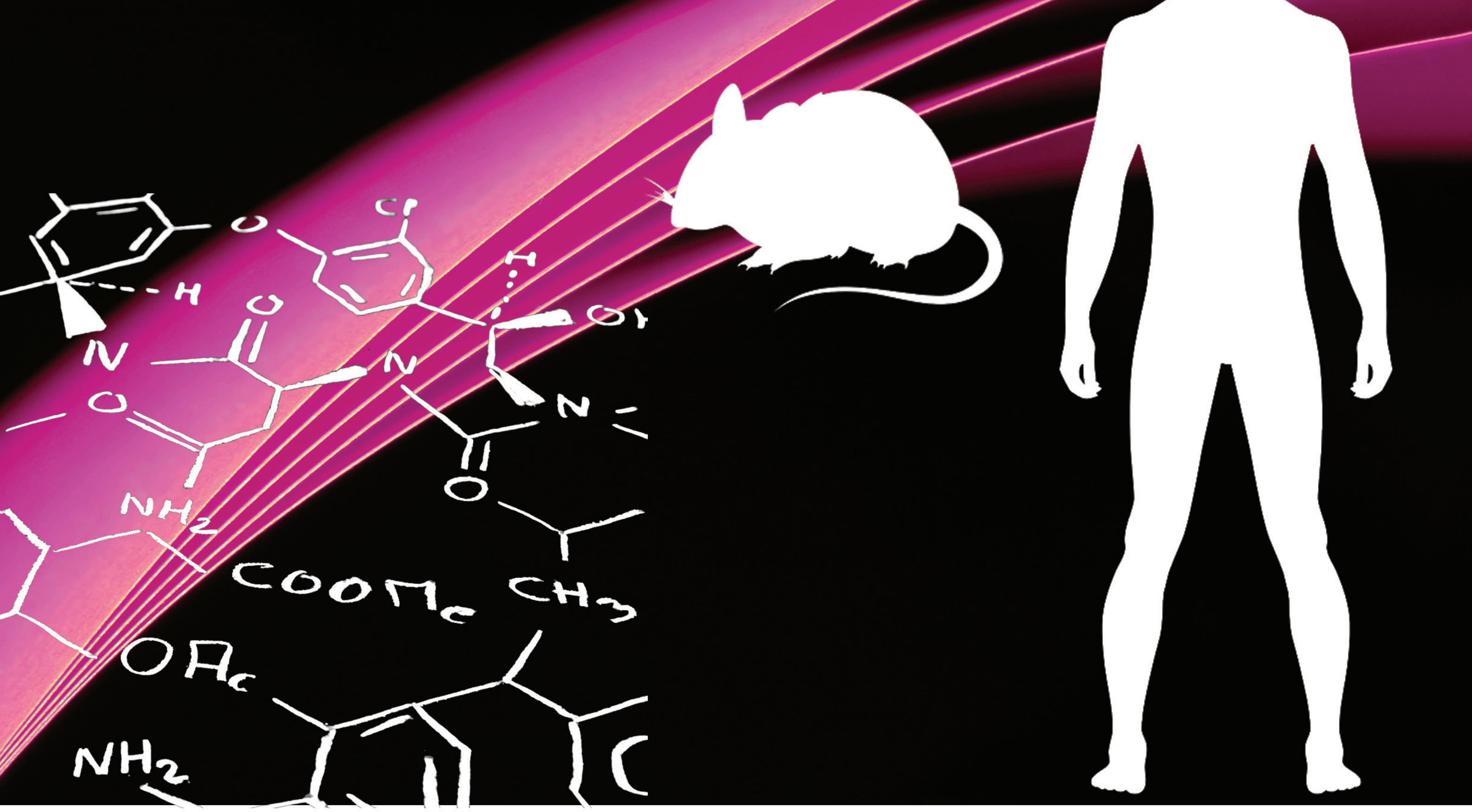

BRITISH PHARMACOLOGICAL SOCIETY

ASPET
Transforming Discoveries into Therapies

WILEY

PRP Pharmacology Research & Perspectives
Open Access

### Aims and Scope

*Pharmacology Research & Perspectives* invites authors to submit research articles and invites submission of several types of special articles. These are:

1. Target validation - publication of negative findings
   - including preclinical papers that show a hypothesis is incorrect
   - or papers on drugs that have failed in early clinical development
2. Drug discovery reviews
   - strategy, hypotheses and data resulting in a successful therapeutic drug
3. Frontiers in translational medicine
   - drug and target validation for an unmet therapeutic need
4. Pharmacological hypotheses
   - reviews that are oriented to inform a novel hypothesis

*Pharmacology Research & Perspectives* publishes papers submitted directly to the journal and those referred from the prestigious journals of BPS and ASPET.

For further information, see the journal's full scope statement available at http://onlinelibrary.wiley.com/journal/10.1002/(ISSN)2052-1707/homepage/ProductInformation.html

### Open Access and Copyright

All articles published by *Pharmacology Research & Perspectives* are fully open access: immediately freely available to read, download and share. All *Pharmacology Research & Perspectives* articles are published under the terms of a Creative Commons Attribution License. Copyright on any research article in a journal published by *Pharmacology Research & Perspectives* is retained by the author(s). Authors grant Wiley a license to publish the article and identify itself as the original publisher. Further information about copyright and the choice of open access licenses can be found at http://www.wileyopenaccess.com/details/content/12f25db4c87/Copyright–License.html.

### Purchasing Print Reprints

Print reprints of Wiley Open Access articles can be purchased from corporatesales@wiley.com.

### Disclaimer

The Publisher and Editors cannot be held responsible for errors or any consequences arising from the use of information contained in this journal; the views and opinions expressed do not necessarily reflect those of the Publisher and Editors, neither does the publication of advertisements constitute any endorsements by the Publisher and Editors of the products advertised.

Wiley Open Access articles posted to repositories or websites are without warranty from Wiley of any kind, either express or implied, including, but not limited to, warranties of merchantability, fi tness for a particular purpose, or non-infringement. To the fullest extent permitted by law Wiley disclaims all liability for any loss or damage arising out of, or in connection with, the use of or inability to use the content.

For submission instructions, subscription, and all the latest information, visit onlinelibrary.wiley.com/journal/10.1002/(ISSN)2052-1707

### Editor-in-Chief

Michael F. Jarvis, PhD, FBPhs
*Chicago, USA*

### Deputy Editors-in-Chief

Jennifer Martin, FRACP, PhD
*Newcastle, Australia*

Ross Corriden
*San Diego, USA*

### Regional Editors

Regional Editor for the Americas
Federico Monczor
*Buenos Aires, Argentina*

Regional Editor for Europe
Beáta Sperlágh
*Budapest, Hungary*

### Senior Editors

Ross Corriden, *San Diego, USA*
Bhagwat Prasad, *Spokane, USA*
Scott Bitner, *Indiana, USA*

### Reviewing Editors

Leon Aarons
*Manchester, UK*
Richard Amison
*London, UK*
Alakananda Basu
*Denton, USA*
Scott Bitner
*Plymouth Meeting, USA*
Stefan Böhm
*Vienna, Austria*
Robyn Brown
*Melbourne, Australia*
Ioannis Charalampopoulos
*Crete, Greece*
Margaret Cunningham
*Glasgow, UK*
Brian Enright
*Chicago, USA*
Ferdinando Estrada
*Buffalo, USA*
Natalia C. Fernández
*Buenos Aires, Argentina*

Ryoichi Fujiwara Little
*Rock, USA*
Clare Guilding
*Newcastle, UK*
Christian Höcht
*Buenos Aires, Argentina*
Dana Hutchinson
*Melbourne, Australia*
Peter Jackson
*Sheffield, UK*
Jianguo Ji
*Chicago, USA*
Hiroshi Katsuki
*Kumamoto, Japan*
Anurag Kuhad
*Chandigarh, India*
Dilip Kumar Singh
*Washington State University, USA*
John Lazo
*Charlottesville, USA*
Chih-Hung Lee
*Chicago, USA*

Jian-Hui Liang
*Beijing, China*
Anastassios Lymperopoulos
*Fort-Lauderdale-Davie, USA*
Qing Ma
*Buffalo, USA*
Graham A. Mackay
*Melbourne, Australia*
Akira Nishiyama
*Takamatsu, Japan*
Terence Pang
*Melbourne, Australia*
Jeff Paul
*Evanston, USA*
Liz Rosethorne
*Nottingham, UK*
Daniel Scott
*Melbourne, Australia*
Elizabeth Sharlow
*Charlottesville, USA*
Hisashi Shirakawa
*Kyoto, Japan*

### Social Media Coordinator

Stephanie Davis
*Lexington, USA*
